# Investigation of Hypoglycemic Peptides Derived from Conserved Regions of adMc1 to Reveal Their Antidiabetic Activities

**DOI:** 10.1155/2021/5550180

**Published:** 2021-03-09

**Authors:** Hafiza Salaha Mahrosh, Rizwan Mehmood, Shazia Anwer Bukhari, Gulnaz Afzal, Rawaba Arif

**Affiliations:** ^1^Department of Biochemistry, Government College University Faisalabad, Pakistan; ^2^Department of Zoology, The Islamia University of Bahawalpur, Bahawalpur, Pakistan

## Abstract

Diabetes mellitus is the most common chronic disorder and leading cause of renal, neurological, and gastrointestinal manifestations in developed and developing countries. Despite of many drugs and combinational therapies, the complications of diabetes are still listed due to severe consequences of those drugs. In past few years, plant-derived drugs draw special attention due to their higher efficacy and fewer side-effects. *Momordica charantia* also known as bitter melon is referred as an antidiabetic and hypoglycemic plant in native populations of Asia and East Africa. In current study, an in silico approach was used to evaluate the interactions and binding patterns of plant-derived peptides devised from a hypoglycemic protein adMc1 of *M. charantia* as potential inhibitor of DPP-IV, SGLT1, and GLUT2 receptor proteins. The study has described a novel approach to investigate hypoglycemic peptides to cure diabetes. A total of eighty tetra-, penta-, and hexapeptides were devised from conserved regions of adMc1 homologs. The molecular docking approach using MOE software was employed to reveal inhibiting potentials of devised peptides against three selected proteins. Out of 30 shortlisted ligands six peptides (i.e. SMCG, DECC, TTIT, RTTI, ARNL and TVEV) accomplished the criteria of being good drug candidates against selected receptor proteins following the drugability assessment test. The overall results are acceptable on the basis of ADMET profiling for being good drug candidates against selected proteins.

## 1. Introduction

Diabetes mellitus (DM) is a heterogenous group of chronic metabolic disorders associated with irregular glucose homeostasis and results in elevated level of blood glucose and insulin resistance [1]. The rapid increase in diabetes cases is estimated to increase 4.4% in 2030 among all age groups [2]. Currently, there are many synthetic hypoglycemic drugs available in market including sulfonylureas, incretins, *α*-glucosidase inhibitors, dipeptidyl peptidase-IV (DPP-IV) inhibitors, sodium-dependent glucose transport proteins (SGLTs) inhibitors, and thiazolidinediones but they result in severe consequences. Therefore, owing to severe side-effects of these drugs, there is a need of new class of drugs with much potency and efficacy [3].

The potential target proteins in diabetes include DPP-IV, SGLTs, glucose transporters (GLUT), *α*-glucosidase inhibitors, and peroxisome proliferator-activated receptors [4]. DPP-IV or adenosine deaminase binding protein is a serine exopeptidase predominantly expressed on surface of cells. DPP-IV selectively involves in N-terminal dipeptide cleavage from a variety of substrates including cytokines, growth factors, and incretin hormones [5]. Since the approval of DPP-IV inhibitors, their importance has been raised clinically to cure DM [6]. DPP-IV inhibitors are a class of oral diabetic drugs commonly used as stable drug candidates which have prospectively been designed using therapeutic agent's strategy focused on deep study on mode of action of incretin peptides [7].

In type 2 diabetes mellitus (T2DM) patients, the insulin resistance leads towards elevated level of glucose and the activation of incretins. Incretins are group of metabolic gut peptides secreted from enteroendocrine cells into the blood after intake of nutrients. Incretins lower the glucose level by stimulating the induction of insulin from islets of Langerhans and also inhibits the glucagon production. Glucagon-like peptide-1 (GLP-1), glucagon-like peptide-2 (GLP-2), and glucose dependent insulinotropic peptide/gastric inhibitory polypeptide (GIP) are two examples of incretin hormones [8].

SGLTs are glucose transporters found in six isoforms (i.e., SGLT1, SGLT2, SGLT3, SGLT4, SGLT5, and SGLT6) and have been found to be scattered across the human body. Among these six isoforms, SGLT1 and SGLT2 are well known and extensively investigated in diabetes. Studies have showed that selective inhibition of SGLT1 can slow postprandial gut uptake of glucose and increase plasma levels of GLP-1 and GIP in healthy volunteers [9]. Glucose transporters are proteins widely distributed in body cells and facilitate in the maintenance of blood glucose level in the human body [2]. Among all the glucose transporters, GLUT2 plays a bidirectional role in specific transportation of glucose in the hepatocytes and absorption and reabsorption of glucose from the enterocytes and the renal tubule particularly [10]. GLUT2 is a sugar carrier that sustains power generation in the cell but can also serve as a receptor for extracellular glucose. GLUT2 is regarded as a competent target in treatment of DM due to its major role in glucose homeostasis.

In developing countries, the plant-based compounds have been used as drugs in 75-80% population to cure various diseases [11–13]. Peptides are generated from specific proteins and then consumed as food ingredients. The bioavailability of these peptides depends on absorption, distribution, metabolism, excretion, and toxicity- (ADMET-) based parameters to reach the target organ [14]. Currently, there are many hypoglycemic drugs in the market but these are correlated with multiple gastrointestinal and renal disorders. In silico targeting of different glucose transporters explores new ways in the management of type 2 diabetes mellitus. In this study, the adMc1 protein of medical plant *Momordica charantia* was used to prepare ready-to-dock library of 80 ligands. This includes the in silico approach to target DPP-IV, SGLT1, and GLUT2 to gain the structural insight of binding patterns of different ligands with receptor proteins. The main objective of this study was to evaluate the insulin-like activity of *Momordica charantia* derived peptides using in silico methods and to explore the best inhibitors of DPP-IV, SGLT1, and GLUT2 receptor proteins among prepared ligands.

## 2. Materials and Methods

### 2.1. Retrieval of the 3D Structures of Receptor Proteins

In this study, the 3D structures of three target molecules were employed to reveal hypoglycemic activities of different peptides which were companionable with the properties of the target binding site in the molecular docking study. The three-dimensional structures of human dipeptidyl peptidase-IV (PDB ID: 4A5S) and sodium-dependent glucose cotransporter (PDB ID: 3DH4) were retrieved from the RCSB Protein Data Bank (https://www.rcsb.org/).The protein sequence of GLUT2 was retrieved from the protein database of NCBI (accession number: P11168.1). The 3D structure of GLUT1 (PDB ID: 5EQG) was used as a template to predict the 3D structure of the GLUT2 protein. MODELLER 9.21 was used for homology modeling, and the best model was selected as a receptor protein for further analysis [15, 16].

### 2.2. Ligand Selection and Receptor Optimization

The protein sequence of the adMc1 protein from *Momordica charantia* was retrieved from the protein database of NCBI (accession No: CDG50933.1). MEME Suite was used to predict motifs from the selected protein homologs [17, 18]. Tetra-, penta-, and hexapeptides were devised from the predicted motifs, and their 3D structures were obtained using ChemSketch in MOL format [19]. Optimization of receptor was done by removing water molecules, addition of hydrogen atoms, energy minimization, and 3D protonation for the perfect and accurate docking. The minimized structures were then docked against devised peptides.

### 2.3. Molecular Docking

The prediction of the active site is the most crucial step in in silico drug discovery as it has a direct impact on the reliability of the results. Molecular docking of 80 ligands was performed at the active site of DPP-IV, SGLT1, and GLUT2 receptor proteins separately. Site finder tool of Molecular Operating Environment (MOE) was used to predict active sites in the selected receptor proteins. The parameters (i.e., rescoring 1: London dG, retain: 10, refinement: force field and rescoring 1: London dG, retain: 10) were set to their default to calculate the interactions of ligands with the binding residues of receptor proteins. All devised peptides were docked against the receptor proteins using MOE. Most appropriate interactions and bindings between ligands and receptor proteins were selected on the basis of the best *S*-score, root mean squared deviation (RMSD), and energy validation rankings.

### 2.4. Drug Scan and ADMET Profiling

The drug-likeness properties were determined following the Lipinski's rule of five using SwissADME [20]. The Lipinski's rule of five is based on five parameters (i.e., molecular mass: ≤500 Dalton, molar refractive index: 40-130, partition coefficient (log *P*): ≤5, hydrogen bond donors: <5, and hydrogen bond acceptors: <10). Only the molecules that accomplish all these parameters could be accepted as potential drug candidates. The online bioinformatic server admetSAR was used for ADMET-based chemical screening of selected drug candidates [21].

## 3. Results

### 3.1. Devising Tetra-, Penta-, and Hexapeptides from adMc1

The MEME Suite was used to explore five motifs in ten selected homologs of the adMc1 protein. The motifs were used to devise tetra-, penta-, and hexapeptides to be docked as ligands against DPP-IV, SGLT1, and GLUT2.

### 3.2. Molecular Docking

Molecular docking provides discernment into structural interactions of inhibitors with the receptor proteins. The prepared library of 80 peptides devised from the adMc1 protein of *M. charantia* was docked against DPP-IV, SGLT1, and GLUT2 receptor proteins using the docking algorithm of MOE software. MOE aligned the suitable confirmations and ranked the ligands based on four factors (i.e., the binding pocket with minimum energy structure, strength of hydrogen bond, root mean squared deviation value, and *S*-score). The results of computational docking showed the potency of adMc1 peptides as good inhibitors of DPP-IV, SGLT1, and GLUT2. In this study, three different receptor proteins were selected due to their major roles in diabetes and upregulation of glucose homeostasis. Top 10 conformations were selected in each analysis based on binding patterns and energy validations.

### 3.3. Interaction Analysis

For the receptor protein DPP-IV, top 10 conformations have been selected based on their structural interactions and *S*-scores. In literature, Glu205, Glu206, Tyr547, and Tyr662 have been reported as major amino acids of catalytic pocket of DPP-IV [22]. In this study, the selected top 10 peptides also showed interactions with these reported amino acids (Table 1). The peptide MRGID showed the best interaction (binding score: -17.8389) with the receptor protein, and Glu206, Tyr547, Arg356, and Pro550 were found to be the leading interactive residues in these interactions. All the remaining ligands also showed strong interactions with Glu205, Glu206, Tyr547, and Tyr662 which are also the active amino acid residues of DPP-IV. These ligands have been revealed as maximum pocket shareholders by interacting with the active amino acids of catalytic triad.

The protein sodium-dependent glucose cotransporter 1 (SGLT1) is a major contributor in the reabsorption of glucose in proximal tubules of nephrons. The binding pocket of the SGLT1 receptor protein contains Ser372 and Ser368 as main interacting amino acids. Out of 80 prepared ligands in this study, the top 10 ligands with best *S*-scores and interactions with these active amino acids were selected (Table 2). The peptides EEQRQA, FDEC, and ITTVE with docking scores of -21.0223, -21.0722, and -20.0948 showed strong interactions with Ser372 and Ser368.

Glucose transporters play major role in renal and intestinal glucose absorption and reabsorption. In current study, the Lys288 showed strong interactions with the GLUT2 protein and revealed as a major interacting amino acid. Among the selected ligands, the peptide REEQR with *S*-score of -17.4161 exhibited potential of being a good drug candidate with the best binding affinity with Lys288, Glu286, Ser284, Arg124, and Ser112. The remaining ligands could also be foremost inhibitors of GLUT2 with the best binding affinity and *S*-scores and could be potential drug candidates (Table 3).

### 3.4. Drugability and ADMET Profiling

**Figure 1:**
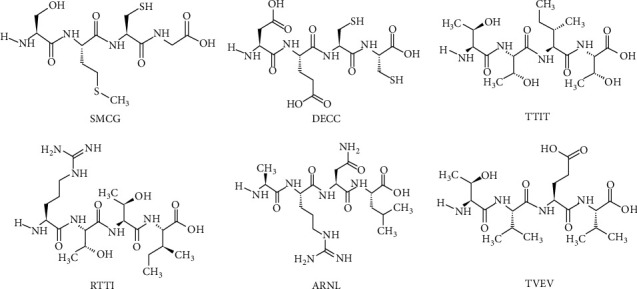
Structures of best selected peptides.

**Figure 2:**
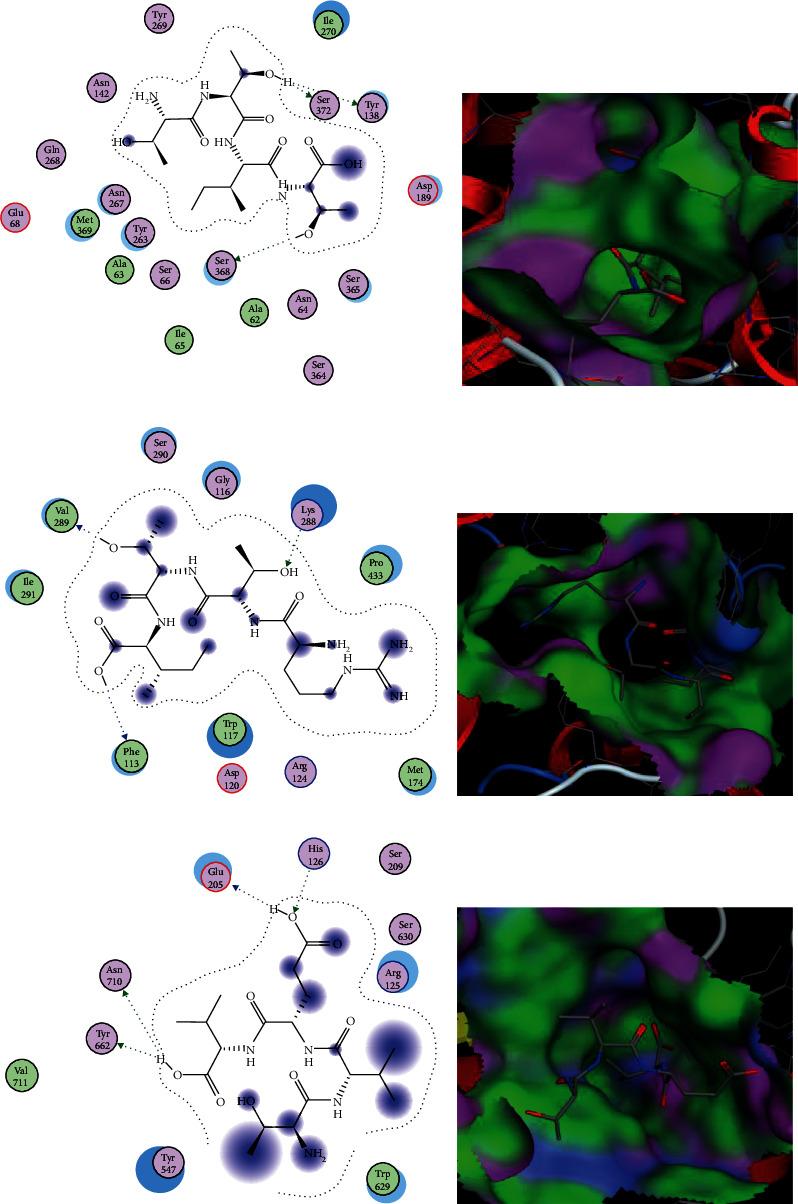
Interactions (on left) and binding patterns (on right) of best selected peptides. (a, b) Interactions and binding patterns of TTIT with SGLT1. (c, d) Interactions and binding pattern of RTTI with GLUT2. (e, f) Interaction and binding pattern of TVEV with DPP-IV, respectively.

The Lipinski's rule of five evaluates the drug-like properties of proposed drug candidates based on five parameters. The rule illustrates the drugability and behavior of selected drug candidate as it distinguishes the drug-like and nondrug-like nature of biologically active ligands. Only the molecules that accomplish all these parameters could be accepted as potential drug candidates. In this study, total 30 ligands have been shortlisted on the bases of their best interactions with active amino acids, *S*-scores, and energy validations. Out of 30 selected ligands used in this study, three tetrapeptides (SMCG, DECC, and TTIT) were found to be effective inhibitors of SGLT1, two tetrapeptides (RTTI and ARNL) were found effective inhibitors of GLUT2, and one tetrapeptide (TVEV) was found to be an efficient inhibitor of DPPI-IV as all these peptides only violated one parameter of Lipinski's rule of five (Table 4). The structures of these six peptides are given in Figure 1. On the basis of Lipinski's rule, these peptides are expected for having reasonable oral bioavailability. The interactions and binding patterns of best peptides each against SGLT1 (i.e., TTIT), GLUT2 (i.e., RTTI), and DPP-IV (i.e., TVEV) have been shown in Figure 2.

For further validations of drug-like behavior of selected ligands, all six ligands were subjected to admetSAR server for assessment of five parameters of ADMET profiling (absorption, distribution, metabolism, excretion, and toxicity). From the results, it has been revealed that all the selected peptides are non-AMES toxic and noncarcinogens (Table 5). The overall results of ADMET drug scanning of these lead ligands were tolerable, and these peptides could be accepted as efficient drug candidates against selected receptor proteins.

## 4. Discussion

Diabetes mellitus is a heterogeneous group of diseases characterized by irregular glucose homeostasis and chronic hyperglycemia due to flaw in insulin secretion and activity [23]. Type 1 diabetes mellitus (insulin dependent) and type 2 diabetes mellitus (noninsulin dependent) are referred as two peculiar forms of diabetes. Type 1 or insulin dependent DM is characterized by total destruction of pancreatic *β*-cells and shutting down the glucose metabolism. The type 2 or noninsulin-dependent diabetes is reported as the most common form due to abnormal insulin secretion and insulin resistance. In type 2 DM, the continuous exposure of body to glucose causes the severity of the disease leading to neurological, gastrointestinal, renal, and cardiovascular syndromes [24]. A vast variety of natural compounds have been reported with medicinal perspectives for the treatment of various diseases [25]. In this study, tetra-, penta-, and hexapeptides (80 in total) were devised from the adMc1 protein of *Momordica charantia*, and their interactions with different receptor proteins that play role in glucose homeostasis were checked through the molecular docking approach. Molecular docking is an elaborative method to evaluate the best possibilities of ligand interactions against different targets for in silico drug designing [26].

The balancing of glucose homeostasis, maintenance of hyperglycemic state to normal level, and shutting of the enzymes that cause irregular glucose homeostasis are the possible ways to control diabetes. Over the past few years, many conventional drug therapies have been in practice but the outcomes are undesirable due to poor efficacy and different side-effects [27]. Thus, scientists are moving towards the use of natural bioactive compounds as antidiabetic agents. The extensive literature survey reveals a large set of targets such as DPP-IV, sodium glucose transporters (SGLTs), *α*-glucosidase, protein tyrosine phosphatase 1B (PTP1B), glucose transporters (GLUT), and G protein-coupled receptors [28–30].

In this study, DPP-IV, SGLT1, and GLUT2 have been listed as target receptor proteins due to their major roles in glucose homeostasis. DPP-IV is a serine exopeptidase that is associated with incretin deficiency in type 2 DM. Current combinational drug therapies include oral hypoglycemic agents including metformin, komboglyze, janumet, and juvisync but these drugs result in unsustainable outcomes. Therefore, the need of new drugs with more efficacies and less side-effects is urgent in order to treat DM [27]. Citrus flavonoids were taken as ligands against different target proteins (e.g., glycogen synthase kinases 3*β*, DPP-IV, and glucokinase) [29]. Shen and Lu [29] have reported that the binding pocket of DPP-IV contains Glu205, Glu206, Tyr662, and Ser209 as active amino acids. In this study, Glu205, Ser209, and Tyr662 were also found as active amino acids as they exhibited strong interactions with DPPI-IV with energy validations.

Sodium glucose transporter-1 (SGLT1) is a glucose transporter expressed at the striated border of intestine. In diabetic patients, the overwhelming glucose concentration might be controlled by SGLT1 inhibition [31]. The 3D structure of human SGLT1 was not available; therefore, the 3D structure of SGLT2 from *Vibrio parahaemolyticus* was taken as a template in this study for the homology modeling of human SGLT1. Both isoforms of sodium glucose transporters share about 60% sequence identity and are highly expressed in S1 segment of border brush membrane [31]. In previous studies, Ser372, Ser50, Asn51, Gly53, Ser54, Gly55, His56, Gln271, Ser368, Ser369, Gln433, and Ser368 of SGLTs have been reported as polar active amino acids [32]. In current study, Ser372 and Ser368 were also found among the interacting residues of SGLT1 that showed strong interactions with the selected ligands.

On the bases of drug-likeness, only 6 peptides have been shortlisted (i.e., SMCG, DECC, TTIT, RTTI, ARNL, and TVEV) followed by Lipinski's rule of five. The remaining 24 ligands violated two or more parameters of Lipinski rule of five. The further assessment of these ligands included ADMET drug scanning to prognosticate the probability of success and failure of potential drug candidates. All the selected ligands passed the criteria of drugability. The efforts and purpose of this study were to target the proteins that play roles in glucose homeostasis to overcome the diabetes mellitus. The mutational changes in certain receptor proteins which are involved in the metabolism and regulation of glucose lead towards severe consequences including the onset of prediabetes and diabetes mellitus. Diabetes mellitus is a group of chronic metabolic diseases that leads towards the onset of many other leading disorders. In the market, many hypoglycemic agents have been in practice to cure diabetes and hyperglycemia but the unsustainable results of these agents lead towards their failure. So, the current need of time is to explore the insulin-like peptides from natural sources with maximum potential and less side-effects.

## 5. Conclusion

The current study offers conversational and comprehensive computational study towards the discovery of insulin-like hit ligands, screened from ready-to-dock library of peptides as ligand molecules against the DPP-IV, SGLT1, and GLUT2 receptor proteins. In this study, 80 highly conserved tetra-, penta-, and hexapeptides as leading ligands were devised from the adMc1 protein of *Momordica charantia*, and the in silico approach was applied to check their molecular dynamic validations and ultimately, to explore their antidiabetic potentials. The peptides SMCG, DECC, TTIT, RTTI, ARNL, and TVEV provided valuable results in drug assessment and ADMET drug scanning. Among the 30 ligands selected on the basis of molecular docking studies, these six tetrapeptides passed the drug assessment tests with great ability to be recognized as drug candidates with potential of inhibiting the DPP-IV, SGLT1, and GLUT2 receptor proteins.

## Figures and Tables

**Table 1:** The interactions of top ten devised peptides with DPP-IV as the receptor protein.

Sr. no.	Peptide	*S*-score	Interacting residues
1	MRGID	-17.8389	Glu206, Tyr547, Arg356, Pro550
2	TTVEV	-15.8132	Glu206, Tyr662, Ser209, Arg669
3	LRQQSR	-15.7212	Glu206, Tyr547, Arg356, Ser209, Arg358
4	TVEV	-15.0490	Glu205, Tyr662, Asn710, His126
5	FDECC	-14.6527	Glu206, Tyr547
6	ECCRE	-14.3605	Tyr547, Tyr662, Tyr666, Pro550, Arg358, Ser209
7	MRGIEN	-14.3088	Glu206, Tyr662
8	RCRQ	-13.5417	Tyr547, Val207
9	TTIT	-13.5247	Glu205, Ser209
10	EECR	-13.3807	Tyr662, Ser209

**Table 2:** The interactions of top ten devised peptides with SGLT1 as receptor protein.

Sr. no.	Peptide	*S*-score	Interacting residues
1	EEQRQA	-21.0223	Ser372, Ser368
2	FDEC	-21.0722	Ser372, Ser368, Ser365, Ser364, Tyr138, Tyr263, Asp189, Ala63, Gln268
3	ITTVE	-20.0948	Ser368, Ser365, Ser364, Asn267, Asp189
4	AREEQR	-19.3288	Ser368, Ser365, Tyr138, Ala361, Ala63, Asp189
5	YAYRTT	-18.9325	Ser368, Tyr263, Asn142, Asn267
6	EEQR	-18.5788	Ser372, Ser368, Ser364, Tyr138, Tyr176
7	SMCG	-17.4970	Ser372, Tyr138, Tyr269, Gln428
8	DECC	-17.3894	Ser368, Ser365, Asn267
9	ERCR	-17.1217	Ser368, Asn142, Thr375
10	TTIT	-16.9639	Ser372, Ser368, Tyr138, Asn142

**Table 3:** The interactions of top ten devised peptides with GLUT2 as the receptor protein.

Sr. no.	Peptide	*S*-score	Interacting residues
1	REEQR	-17.4161	Lys288, Glu286, Ser284, Arg124, Ser112
2	YAYRTT	-17.1186	Lys288, Glu282, Arg124
3	ITTVEV	-16.9540	Lys288, Glu286, Ala283, Ser169
4	LEEIA	-15.8995	Lys288, Ser169
5	YLRQ	-14.8063	Lys288
6	RCRQ	-13.5942	Lys288
7	RTTI	-13.5003	Lys288, Phe113, Val289
8	ARNL	-12.9365	Lys288
9	FDEC	-12.8992	Lys288
10	TITTVE	-15.6597	Ser169, Ser112

**Table 4:** Pharmacokinetic parameters important for bioavailability of compounds drug-likeness properties of selected peptides.

Peptides	Target	Molecular properties^†^						
		MW	HBD	HBA	Nrotb	Log *P*	*A*	Violations
SMCG	SGLT1	396.48	6	7	15	-1.86	94.77	1
DECC		468.50	7	10	17	-2.39	107.10	1
TTIT		434.48	8	9	15	-2.06	105.61	1
RTTI	GLUT2	489.57	10	9	19	-2.17	123.16	1
ARNL		472.54	9	8	19	-2.38	118.94	1
TVEV	DPP-IV	446.50	7	9	16	-0.93	109.86	1
FDEC		512.53	7	10	18	-1.40	123.66	2

^†^Molecular properties were calculated using SwissADME an online tool. MW: molecular weight; HBD: number of hydrogen bond donors; HBA: number of hydrogen bond acceptors; nrotb: number of rotatable bonds; log *P*: the logarithm of octanol/water partition coefficient; *A*: molar refractivity.

**Table 5:** ADMET profiling of best selected peptides.

	SMCG	DECC	TTIT	RTTI	ARNL	TVEV
Absorption						
Blood-brain barrier	BBB+	BBB+	BBB+	BBB+	BBB+	BBB+
Human intestinal absorption	HIA-	HIA-	HIA-	HIA-	HIA+	HIA-
Caco-2 permeability	Caco-2-	Caco-2-	Caco-2-	Caco-2-	Caco-2-	Caco-2-
P-Glycoprotein substrate	Substrate	Nonsubstrate	Nonsubstrate	Nonsubstrate	Substrate	Nonsubstrate
P-Glycoprotein inhibitor	Noninhibitor	Noninhibitor	Noninhibitor	Noninhibitor	Noninhibitor	Noninhibitor
Renal organic Cation transporter	Noninhibitor	Noninhibitor	Noninhibitor	Noninhibitor	Noninhibitor	Noninhibitor
Metabolism						
CYP3A4 substrate	Substrate	Nonsubstrate	Nonsubstrate	Nonsubstrate	Substrate	Nonsubstrate
CYP2C9 substrate	Nonsubstrate	Nonsubstrate	Nonsubstrate	Nonsubstrate	Substrate	Nonsubstrate
CYP2D6 substrate	Nonsubstrate	Nonsubstrate	Nonsubstrate	Nonsubstrate	Nonsubstrate	Nonsubstrate
CYP3A4 inhibition	Noninhibitor	Noninhibitor	Noninhibitor	Noninhibitor	Noninhibitor	Noninhibitor
CYP2C9 inhibition	Noninhibitor	Noninhibitor	Noninhibitor	Noninhibitor	Noninhibitor	Noninhibitor
CYP2C19 inhibition	Noninhibitor	Noninhibitor	Noninhibitor	Noninhibitor	Noninhibitor	Noninhibitor
CYP2D6 inhibition	Noninhibitor	Noninhibitor	Noninhibitor	Noninhibitor	Noninhibitor	Noninhibitor
CYP1A2 inhibition	Noninhibitor	Noninhibitor	Noninhibitor	Noninhibitor	Noninhibitor	Noninhibitor
Toxicity						
AMES toxicity	Non-AMES toxic	Non-AMES toxic	Non-AMES toxic	Non-AMES toxic	Non-AMES toxic	Non-AMES toxic
Carcinogens	Noncarcinogens	Noncarcinogens	Noncarcinogens	Noncarcinogens	Noncarcinogens	Noncarcinogens

## Data Availability

The data used to support the findings of this study are available from the corresponding author upon request.
